# BRAFV600E, BANCR, miR-203a-3p and miR-204-3p in Risk Stratification of PTC Patients

**DOI:** 10.3390/biomedicines11123338

**Published:** 2023-12-18

**Authors:** Stefana Stojanović, Sonja Šelemetjev, Ilona Đorić, Jelena Janković Miljuš, Svetislav Tatić, Vladan Živaljević, Tijana Išić Denčić

**Affiliations:** 1 Department of Endocrinology and Radioimmunology, Institute for the Application of Nuclear Energy—INEP, University of Belgrade, Banatska 31b, 11000 Belgrade, Serbia; stefana@inep.co.rs (S.S.); sonja@inep.co.rs (S.Š.); marecko@inep.co.rs (I.Đ.); jelenaj@inep.co.rs (J.J.M.); 2 Institute for Pathology, Faculty of Medicine, University of Belgrade, Doctor Subotic Street 1, 11000 Belgrade, Serbia; static@med.bg.ac.rs; 3 Clinic for Endocrine Surgery, University Clinical Center of Serbia, Pasterova 2, 11000 Belgrade, Serbia; vladanzivaljevic@gmail.com

**Keywords:** papillary thyroid carcinoma, long non-coding RNA, microRNAs, tumor markers, aggressiveness, prognosis

## Abstract

In order to enhance the risk stratification of papillary thyroid carcinoma (PTC) patients, we assessed the presence of the most common mutation in PTC (BRAFV600E) with the expression profiles of long non-coding RNA activated by BRAFV600E (BANCR) and microRNAs, which share complementarity with BANCR (miR-203a-3p and miR-204-3p), and thereafter correlated it with several clinicopathological features of PTC. BRAFV600E was detected by mutant allele-specific PCR amplification. BANCR and miRs levels were determined by quantitative RT-PCR. Bioinformatic analysis was applied to determine the miRs’ targets. The expression profile of miR-203a-3p/204-3p in PTC was not affected by BRAFV600E. In the BRAFV600E-positive PTC, high expression of miR-203a-3p correlated with extrathyroidal invasion (Ei), but the patients with both high miR-203a-3p and upregulated BANCR were not at risk of Ei. In the BRAFV600E-negative PTC, low expression of miR-204-3p correlated with Ei, intraglandular dissemination and pT status (*p* < 0.05), and the mutual presence of low miR-204-3p and upregulated BANCR increased the occurrence of Ei. Bioinformatic analysis predicted complementary binding between miR-203a-3p/204-3p and BANCR. The co-occurrence of tested factors might influence the spreading of PTC. These findings partially describe the complicated network of interactions that may occur during the development of PTC aggressiveness, potentially providing a new approach for high-risk PTC patient selection.

## 1. Introduction

Thyroid carcinoma is the most common form of endocrine malignancy, with an increasing incidence in recent years [1]. Papillary thyroid carcinoma (PTC), its main form, is generally a highly curable and indolent malignancy with an excellent prognosis, but 5–10% of the patients who relapse do not respond to conventional therapies and may die from the disease [2]. The challenge in thyroid pathology is the identification of these high-risk PTC patients, among the PTC patients with less aggressive forms of carcinoma, in order to accordingly personalize the treatment.

PTC is characterized by a great diversity of histopathological features which are the consequence of a great diversity of the genetic and epigenetic variations of the tumor [3]. The most common genetic changes in PTC are mutations in B-type RAF kinase (BRAF) of the RAS/BRAF/mitogen-activated protein kinase signaling pathway [4]. Among these, BRAFV600E mutation, which accounts for >95% of all BRAF mutations, is the most frequently reported BRAF mutation in PTC. It has been reported to be present in about 29–83% of cases of PTC [4,5]. The presence of the mutation causes the MAPK/ERK pathway to be constitutively activated, which in turn promotes the growth and proliferation of cancer cells. While a majority of studies showed a significant association of the BRAFV600E mutation with one or more conventional high-risk clinicopathological characteristics and/or poor prognosis of patients with PTC, some controversial findings were also reported by other investigators [4,5,6]. To improve the treatment of this disease, it is crucial to comprehend the other molecules that regulate the development and progression of PTC.

The Human Genome Project discovered that more than 90% of the genome is composed of non-coding RNAs (ncRNAs), RNAs that are transcribed from the genome, but they are not translated into proteins. Instead, ncRNAs regulate the expression of other genes at all molecular levels, including chromatin and gene splicing, transcription and posttranscriptional events [7]. Consequently, they are involved in many biological processes and diseases, including cancer development [8]. NcRNAs that are differentially expressed in thyroid cancer tissues compared with normal thyroid tissues are relevant to the diagnosis and prognosis of thyroid cancer, and some of them may be potential new therapeutic targets [8,9]. NcRNAs can be classified according to their size into long ncRNAs (lncRNAs) and short ncRNAs.

LncRNAs are a class of ncRNAs containing more than 200 nucleotides [10]. To date, only a small fraction of lncRNAs have been discovered, and the importance of lncRNAs in tumorigenesis, metastasis and tumor progression needs further investigation. BRAF-activated lncRNA (BANCR) is a 693 bp long transcript activated by the BRAFV600E mutation and with deregulated expression in several types of human carcinomas, including PTC [11,12,13,14,15]. It has been shown that the level of expression of BANCR influences the development and progression of PTC, but the results are still controversial [13,14,15,16].

MicroRNAs (miRs) are a subgroup of short ncRNAs (19–23 nucleotides in length) that control the expression of genes in various biological processes, such as cell differentiation, proliferation and apoptosis. Due to its suppressive effect, a certain regulatory mechanism involving miR may lead to the downregulation of tumor suppressor genes and/or the upregulation of oncogenes [17]. Alterations in miRs expression have been reported as important regulators of thyroid cancer progression and development [18]. In addition, researchers have recently discovered a new regulatory mechanism for lncRNAs that allows them to function as competitive endogenous RNAs by competing with other transcripts for shared miRs or acting as a sponge for miRs that bind lncRNA in a complementary manner [19]. Therefore, lncRNAs may play a posttranscriptional regulatory role in the distribution of miRs to their targets. MiR-203a-3p and miR-204-3p are newly discovered miRs with deregulated expression in PTC and with a rather unknown biological function. Studies have shown that miR-203a-3p and miR-204-3p are often downregulated in thyroid cancer, which can lead to increased expression of oncogenes and decreased expression of tumor suppressor genes [20,21,22]. Furthermore, a study on colorectal cancer cells found that BANCR works as a miR-203 sponge [23], while a study on melanoma cells discovered that miR-204 is a direct target of BANCR [24]. In addition, BANCR negatively modulated miR-204-3p expression via direct assimilation in the retinoblastoma cell line [25].

In this article, we analyzed the factors shown to promote the abnormal growth and proliferation of thyroid cancer cells through diverse molecular mechanisms. We assessed the mutual expression profiles of miRs (miR-203a-3p or miR-204-3p) and lncRNA BANCR in PTC tissue samples, along with the occurrence of the BRAFV600E mutation, and thereafter correlated it with various clinicopathological factors of PTC patients. The results call attention to the complex interactions that may occur during the process of PTC spreading.

## 2. Materials and Methods

### 2.1. Tissue Samples

A total of 76 pairs of PTC tumors and adjacent normal thyroid tissue were obtained from patients who had undergone surgical resection at the Clinic for Endocrine Surgery, Clinical Center of Serbia, Belgrade. The resected tissue samples were immediately frozen in liquid nitrogen and stored at −80 °C for further use. Informed consent was obtained from all patients who participated in the study for the use of excess biological material for investigational purposes. This study is in accordance with the Declaration of Helsinki and was approved by the Ethics Committee of the Faculty of Medicine, University Clinical Center of Serbia. Histopathological diagnosis was made by a pathologist according to established cytohistopathological criteria [26].

Clinicopathological information regarding patient age and sex, dimension of the tumor, presence of lymph node metastases (Lnm), intraglandular dissemination (ID), the occurrence of extrathyroidal extension (Ei), and distant metastases presence was obtained through a comprehensive evaluation of pathology reports. The grading of carcinomas followed the pathologic tumor–node–metastasis (pTNM) staging system established by the American Joint Committee on Cancer (AJCC) [27]. Additionally, the degree of tumor infiltration (DTI) was reassessed according to previously published classification [28].

Among the patients included in this study, there were 58 females (76.3%) and 18 males (23.6%), with ages ranging from 14 to 89 years at diagnosis (52.9 ± 16.3, mean ± SD). Tumor dimensions ranged from 2 to 120 mm (29.9 ± 16.7 mm, mean ± SD), while 12 cases exceeded 40 mm (15.6%). Within the cohort of PTC cases, there were 16 cases of the classical variant, 29 cases of the follicular variant, 12 cases characterized by mixed histopathological patterns, representing follicular and classical areas in diverse percentages, and 19 cases with areas of rare histologic variants of PTC, including tall cell, solid, trabecular, Warthin-like, clear cell and oxyphilic subtypes. ID was observed in 40 (51.9%), Lnm in 14 (18.2%) and Ei in 21 (27.3%) cases of primary PTC. No patient had a known distant metastasis in our sample series. According to the previously published data [2,29], patients’ age ≥ 45 years, tumor size >40 mm, Ei or distant metastasis presence were regarded as being poor predictive factors of survival, while the presence of Lnm signified a risk of recurrence in patients with PTC.

### 2.2. RNA and DNA Isolation

The equivalent of 100 mg of frozen PTC tissue was homogenized using TissueLyser LT (Qiagen GmbH, Hilden, Germany).

For the isolation of RNA, homogenization was performed at 50 Hz for 5 min. Afterwards, total RNA was extracted from the samples using TRIzol reagent (Invitrogen, Carlsbad, CA, USA) according to the manufacturer’s instructions. RNA concentration was quantified using an Epoch microplate spectrophotometer (BioTek, Winooski, VT, USA).

For the isolation of DNA, homogenization was performed at 20 Hz for 2 min, followed by overnight incubation with 500 µL of digestion buffer (50 mmol Tris, pH 8.0; 100 mmol EDTA; 100 mmol NaCl; 1% SDS) and 50 µg of Proteinase K (Sigma-Aldrich, Taufkirchen, Germany) at 55 °C. Samples were then treated with 300 µL of 6M NaCl for protein precipitation and centrifuged at 4 °C. After treating the supernatant with an equal volume of ice-cold isopropanol, the precipitated DNA was subjected to washing with ice-cold 70% ethanol, followed by drying and subsequent dissolution of DNA in 30 µL of nuclease-free water. The DNA concentration was determined using a Nano Vue spectrophotometer (GE Healthcare, Buckinghamshire, UK).

### 2.3. BRAF Mutational Analysis

We performed mutant allele-specific PCR amplification (MASA) to verify the presence of the BRAF V600E point mutation. Two forward primers (for wild type and the BRAFV600E mutation) and one reverse primer were used as described previously [30]. Their sequences are listed in Table 1.

### 2.4. Reverse Transcription and Quantitative Real-Time PCR for BANCR

Reverse transcription and quantitative real-time PCR for BANCR are described in detail in our previously published paper [16]. Primers’ sequences are shown in Table 1. The relative expression of BANCR was calculated as the relation of mRNA of BANCR expression versus the expression of mRNA of GAPDH in the same tissue sample (2^−ΔCt^ method) (BANCR relative expression). The fold change in BANCR was calculated as the relationship of BANCR relative expression in malignant vs. BANCR relative expression in adjacent NMT (2^−ΔΔCt^ method). BANCR was declared as upregulated if its level of expression was higher in PTC than in adjacent NMT (fold change > 1); and if the BANCR level of expression was lower in PTC than in adjacent NMT, it was declared as downregulated (fold change < 1).

### 2.5. Reverse Transcription and Quantitative Real-Time PCR for miR-203a-3p and miR-204-3p

Reverse transcription and quantitative real-time PCR for miR-203a-3p and miR-204-3p are described in detail in our previously published paper [22]. Table 1 provides information about the sequences of stem-loop primers used in reverse transcription reactions, as well as the sequences of the primers used in qPCR. MiRs relative expression was achieved by normalizing its expression to the U6 mRNA expression (2^−ΔCt^ method). In order to determine the miRs fold change, the 2^−ΔΔCt^ method was applied. miR were assembled into high and low expression groups based on their median value; the cases with a relative expression higher or equal to the median value were declared as high expression ones and the cases with a relative expression lower than the median value were declared as the low expression ones.

### 2.6. Bioinformatic Analysis

Bioinformatic analysis was performed to establish sequence complementarity of miRs and lncRNA, as well as for miR target prediction. Freely available bioinformatics tools were used for miRs’ target prediction (BiBiServ—the Bielefeld University Bioinformatics Server, http://bibiserv.cebitec.uni-bielefeld.de (accessed on 30 May 2023) and miRDB—MicroRNA Target Prediction Database, http://www.mirdb.org/ (accessed on 29 November 2019).

### 2.7. Statistical Data Analysis

The normality of the distributions was tested by d’Agostino–Pearson’s test, the statistical test for the distribution type. The statistical significance of differences between groups was determined by *t*-test and Median test. Correlation analyses were evaluated using Spearman’s correlation. For frequency testing, samples were divided into high and low expression groups according to the median of the tested variable. The χ^2^-test was applied to variables whose expected number was equal to or greater than 5. When the expected number was less than 5, Fisher’s exact test was applied. The results were considered significant if the *p*-value < 0.05. Statistical analysis was performed using SPSS (SPSS 16.0, Chicago, IL, USA) and Simple Interactive Statistical Analysis (SISA) software, which allows online statistical analysis. (https://www.quantitativeskills.com/sisa/, accessed on 15 August 2023).

## 3. Results

The results were first tested for the distribution type. According to the statistical analysis, the expression of tested ncRNAs had no normal distribution (*p* < 0.05), therefore a non-parametric set of tests was further applied.

### 3.1. The Expression of miR-203a-3p and miR-204-3p in the BRAFV600E-Positive and BRAFV600E-Negative PTC

**Figure 1:**
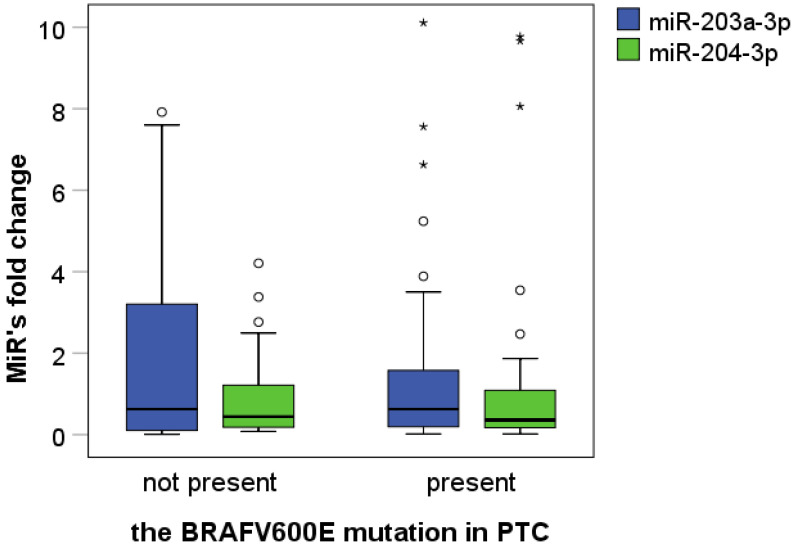
Association of BRAFV600E presence with miR-203a-3p and miR-204-3p fold change. Circles represent the mild outliers and stars represent the extreme outliers.

In our series of 76 PTC samples, there were 55 PTCs with the BRAFV600E mutation (55/76, 72.4%) and 21 BRAFV600E-negative PTCs (21/76, 27.6%). The descriptive statistics of the relative expression levels of tested miRs in the BRAFV600E-positive and BRAFV600E-negative PTCs are given in Table 2. Tested miRs’ fold changes in dependence of the BRAFV600E mutation presence are presented in Figure 1. According to our results, there is no statistical difference in the level of expression of miR-203a-3p/204-3p, or their fold changes, between the BRAFV600E-positive and BRAFV600E-negative PTC (tested by Median test and *t*-test, *p* > 0.05).

### 3.2. Correlation of miR-203a-3p and miR-204-3p Expression with Clinicopathological Parameters of the BRAFV600E-Positive and BRAFV600E-Negative PTC

The correlation of miR-203a-3p and miR-204-3p levels of expression with the clinicopathological parameters of the BRAFV600E-positive and BRAFV600E-negative PTC is given in Table 3.

By splitting up the total PTC sample according to the BRAFV600E mutation presence and correlating it with the unfavorable clinicopathological parameters of the patients, two distinct trends could be noticed: in the BRAFV600E-positive PTC, high expression of miR-203a-3p correlates with Ei and DNI, while in the BRAFV600E-negative PTC, there is a negative correlation of the miR-204-3p level of expression with Ei, ID and pT (*p* < 0.05 for all). In other words, high miR-203a-3p expression in the BRAFV600E-positive PTC (r = 0.282, *p* = 0.037) and low miR-204-3p expression in the BRAFV600E-negative PTC (r = −0.452, *p* = 0.040) correlate with the presence of Ei.

### 3.3. Bioinformatic Analysis and Prediction of Interaction between miR-203a-3p/miR-204-3p and BANCR

**Figure 2:**
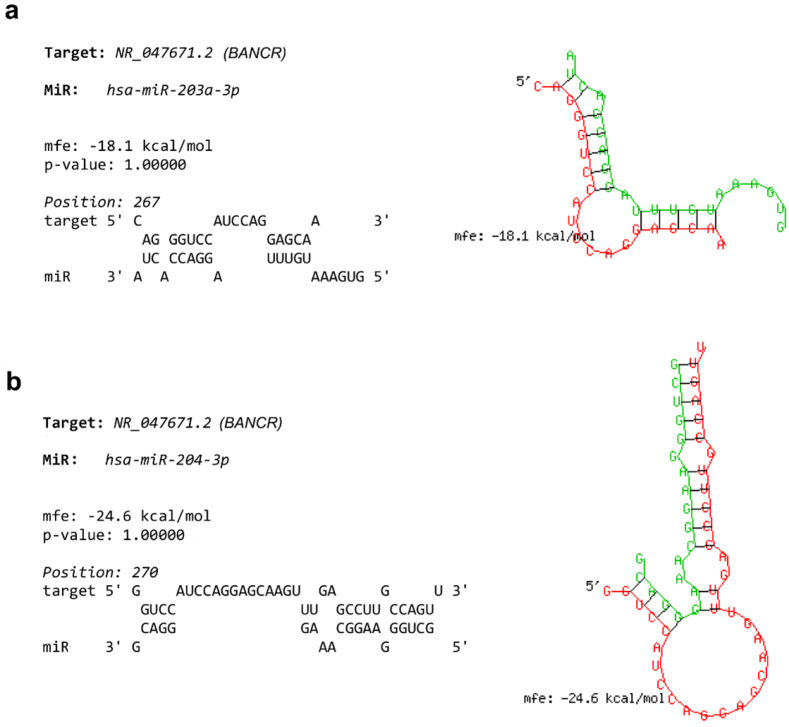
Predicted binding sites between BANCR and tested miRs according to BiBiServ (the Bielefeld University Bioinformatics Server): (**a**) BANCR (red) and miR-203a-3p (green), (**b**) BANCR (red) and miR-204-3p (green).

A growing body of evidence suggests that lncRNAs act as molecular sponges for miRs to exercise their regulatory influence on target miRs. To further investigate the potential interaction of tested miRs in PTC, online prediction websites were employed to test in silico if these miRs might interact with BANCR. The presence of several complimentary sites between miR-203a-3p/miR-204-3p and BANCR was shown by the algorithms, implying that BANCR could interact with miR-203a-3p and miR-204-3p. The analysis predicted complementary binding between miR-203a-3p and BANCR (Figure 2a), as well as between miR-204-3p and BANCR (Figure 2b), indicating that BANCR may sequester miR-203a-3p and miR-204-3p and hence be inhibited by tested miRs.

### 3.4. Mutual Expression of miRs (miR-203a-3p or miR-204-3p) and BANCR with the Occurrence of the BRAFV600E Mutation and Extrathyroidal Invasion of PTC

The association of the combined miR-203a-3p/204-3p level of expression and BANCR deregulation with the presence of Ei in the BRAFV600E-positive and BRAFV600E-negative PTC is shown in Table 4. 

Statistically significant association of the low level of miR-204-3p expression and the upregulation of BANCR with the Ei presence was found only in the BRAFV600E-negative PTC (*p* = 0.033), while the statistically significant association of the high level of miR-203a-3p expression and the upregulation of BANCR with the presence of Ei was found only in the BRAFV600E-positive PTC (*p* = 0.009). As shown in Table 4, in PTC with the BRAFV600E mutation, low miR-203a-3p and downregulated BANCR, the chances for Ei are quite low. On the contrary, if miR-203a-3p expression is high or BANCR is unchanged or upregulated, the BRAFV600E-positive PTC is prone to Ei. Any sample with both high miR-203a-3p and upregulated BANCR did not have Ei in our series of the BRAFV600E-positive PTC cases. On the other hand, in the BRAFV600E-negative PTC, only cases with high miR-204-3p and downregulated BANCR did not have Ei. PTC patients who had either low miR-204-3p or upregulated BANCR were not at high risk of Ei; however, PTC patients who had both low miR-204-3p and upregulated BANCR are.

**Figure 3:**
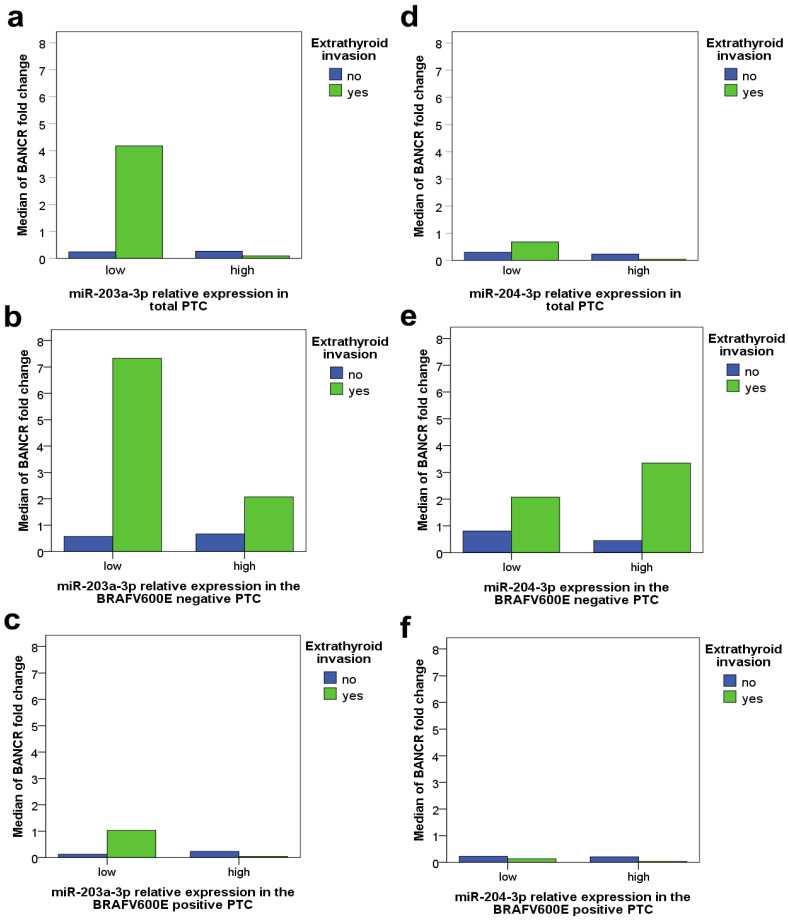
Relationship between BRAFV600E, BANCR, miR-203a-3p and miR-204-3p and extrathyroidal invasion occurrence. Explanation of subfigures (**a**–**f**) is given in the body text of the manuscript above.

The dependency of the occurrence of Ei on the relation of miRs’ level of expression (low/high) and BANCR fold change in the total PTC, the BRAFV600E-positive and BRAFV600E-negative PTC are shown in Figure 3, too. As shown before [16] and as it can be seen from the graphs, there is a difference in the distributions of BANCR deregulation between the total PTC sample and the sample separated on the base of the BRAFV600E mutation presence. It is interesting to notice that while the upregulation of BANCR is associated with Ei occurrence in total PTC, in BRAFV600E-positive PTC, Ei persists in cases with either low miR-203a-3p and unchanged levels of BANCR, on average, or with high miR-203a-3p and significantly downregulated BANCR (Figure 3c). This coincides with the findings presented in Table 4 (and described in the previous paragraph), that the combination of high miR-203a-3p and upregulated BANCR does not lead to Ei in PTC (Figure 3a,c). On the other hand, while lower levels of miR-204-3p expression associate with Ei in total PTC (Figure 3d), in the BRAFV600E-negative PTC, cases with upregulated BANCR also developed Ei, independently of the miR-204-3p level of expression (Figure 3e).

To sum up, the level of miR-203a-3p and miR-204-3p expression does not depend on the BRAFV600E mutation presence in PTC. High miR-203a-3p expression correlates with the presence of Ei in the BRAFV600E-positive PTC. Low miR-204-3p expression correlates with the presence of Ei in the BRAFV600E-negative PTC. There is no BRAFV600E-positive PTC case with Ei, mutually upregulated BANCR and high miR-203a-3p expression.

## 4. Discussion

The biggest difficulty in thyroid cancer diagnosis and therapy is distinguishing high-risk patients from those with a less aggressive form. While the clinical risk stratifications given by the American Thyroid Association are highly helpful in guiding decision making, they may be insufficient in some circumstances, and thus new biomarkers that may help to adapt, optimize and personalize clinical treatment would be advantageous. Since the genetic and epigenetic alterations associated with the pathogenesis of PTC may serve as novel prognostic markers or therapeutic targets, in this work, we investigated the relationship between the clinicopathological features of PTC patients, the mutational status of the most common genetic alteration in PTC (BRAFV600E), the expression levels of long non-coding RNA activated by the BRAFV600E mutation (BANCR), and the expression levels of two miRs that share complementarity with BANCR (miR-203a-3p and miR-204-3p). According to our results, the BRAFV600E mutation, BANCR, and expression of miR-203a-3p and miR-204-3p might jointly contribute to the development and progression of PTC.

In this work, we detected 72.4% BRAFV600E-positive PTC, which is consistent with previous reports that BRAFV600E is present in approximately 29–83% of PTC cases [4,5,6]. This strikingly wide variation in the prevalence of the BRAFV600E mutation reported by different authors can be explained by the different distribution of histological subtypes of PTC, sample size, the patients’ age distribution and variation in detection methods [4]. In addition, although BRAFV600E is associated with the presence of some unfavorable clinicopathological parameters in PTC patients and with increased recurrence and mortality rates, it is not an independent predictor of unfavorable outcomes [6], and the prognostic value of BRAFV600E remains a controversial issue [4,5,6]. Therefore, the relationship between the clinicopathologic features of PTC, BRAFV600E mutational status, and underlying genetic and epigenetic background variations needs further investigation.

LncRNAs are RNA molecules that are transcribed from non-protein-coding sequences in the genome and play important roles in gene regulation at the epigenetic, transcriptional and translational levels, as well as in posttranslational protein modification [7,10]. Consequently, lncRNAs are associated with processes such as tumorigenesis, as well as tumor progression, metastasis and recurrence [8,11]. BANCR is a recently identified lncRNA that is activated by the BRAFV600E mutation [12]. It has been found to be deregulated in various types of human carcinomas, acting as a tumor suppressor in some cases and as a tumor promoter in others [13]. Moreover, in some cancers such as colorectal cancer, hepatocellular carcinoma and papillary thyroid carcinoma, there is no agreement on the expression of BANCR, and its biological function is still unclear [13]. Therefore, additional functional studies in these tissues are needed. For example, in a study of the xenograft model of thyroid cancer, the upregulation of BANCR enhanced tumor growth [31]; in another study on IHH-4 thyroid cancer cells, BANCR enhanced proliferation, prevented apoptosis and G1 arrest, and induced autophagy [15]. We have previously shown that the presence of the BRAFV600E mutation in PTC modulates BANCR expression; BANCR was generally downregulated in PTC with the BRAFV600E mutation, while it was generally unchained or upregulated in PTC without the BRAFV600E mutation [16]. On the other hand, Liao et al. [14] demonstrated that BANCR may operate as a tumor suppressor in PTC. According to the findings, BANCR expression tended to be downregulated in human PTC tissues, while its overexpression reduced PTC cell proliferation and promoted apoptosis, which inhibited metastasis. Decreased BANCR levels correlated with PTC size, the presence of multifocal lesions and advanced PTC stage. Furthermore, PTC tumors with high BANCR levels tended to be more differentiated than those with low BANCR levels. Therefore, BANCR clearly has a role in the incidence and progression of PTC through modifying several tumor-related signaling pathways, including MAPK, but its ultimate effect is likely to be dependent on other regulatory mechanisms. It has been demonstrated that the interaction between lncRNA and miRs influences tumor formation and progression [19]. As a result, the expression levels of target miRs in each tissue may define BANCR’s oncogenic or tumor suppressive activity. However, it has not been investigated as to how this relates to the clinicopathological characteristics of PTC patients.

The expression of miR-203a-3p and miR-204-3p in PTC is analyzed in detail in our previous work [22]. When comparing NMT to primary PTC, there was no statistically significant difference in the expression of miR-203a-3p/miR-204-3p [22]. TCGA analysis corroborated our findings for miR-203a-3p expression, while it demonstrated upregulated values of miR-204-3p expression in PTC compared to non-malignant thyroid tissue [22]. There was a significant correlation between the miR-203a-3p level of expression with the presence of Ei, as well as with higher DTI in PTC, while the level of expression of miR-204-3p in PTC was inversely correlated with its pTNM stage. However, it was found that none of the miRs (miR-203a-3p/miR-204-3p) could be used as independent prognostic factors in PTC [22]. Thus, it appears that the development of aggressive PTC behavior is influenced by some other factors, and in this work, we have tried to elucidate if BRAFV600E and BANCR are parts of that puzzle. According to the findings of the first section of this study, the BRAFV600E mutation has no effect on the fold changes or expression levels of miR-203a-3p and miR-204-3p in the PTC sample. Although the existence of the BRAFV600E mutation does not directly affect the expression levels of miR-203a-3p and miR-204-3p, it might be that different pathways of PTC development are activated, depending on the presence of BRAFV600E mutation and miRs’ levels. Because BANCR fold change is dependent on the presence of BRAFV600E in PTC [16] and as bioinformatics analysis predicted complementary binding of miR-203a-3p and miR-204-3p with BANCR, we attempted to determine if BANCR fold change could be the factor that determines PTC spreading. The results presented in the second section of this study indicate that high miR-203a-3p expression associates with extrathyroidal invasion in BRAFV600E-positive PTC, while low miR-204-3p expression associates with thyroid gland capsule invasion in BRAFV600E-negative PTC. However, in our series of BRAFV600E-positive PTC cases, none of them invaded the thyroid gland capsule with jointly upregulated BANCR and high miR-203a-3p. Furthermore, bioinformatic analysis predicted the complementary binding of miR-203a-3p and BANCR. Consequently, it appears that miR-203a-3p and BANCR may interact, and that the balance of their expressions influences the occurrence of extrathyroidal invasion in PTC. In other words, these data collectively suggest that in BRAFV600E-positive PTC, BANCR may function as a sponge for miR-203a-3p, reducing the possibility of thyroid gland capsule invasion. Similarly, a study on colorectal cancer cells reported that BANCR acts as a miR-203 sponge [23]. Furthermore, Shi et al. [32] revealed that patients with BRAFV600E-positive PTC overexpressed BANCR, and downregulated miR-9 patients are at increased risk of recurrence, thus necessitating earlier surgical treatment and total thyroidectomy in initial surgery. In our study, bioinformatic analysis also predicted complementary binding of miR-204-3p and BANCR, and in our group of BRAFV600E-negative PTC patients, upregulated BANCR and low miR-204-3p levels were associated with the invasion of the thyroid gland capsule. This could imply that, because of the complementary miR-204-3p and BANCR sequences, upregulated BANCR might sponge miR-204-3p. The consequence of such findings might be that BANCR further lowers the levels of miR-204-3p in BRAFV600E-negative PTC patients, which increases the possibility of thyroid gland invasion. Therefore, it seems that BANCR and miR-204-3p might act in a cumulative way on the development of the extrathyroidal invasion of the BRAFV600E-negative PTC. Similar findings were obtained in melanoma and retinoblastoma cells [24,25], but this is the first investigation on thyroid cancer tissue samples (rather than cell cultures). Despite certain limitations of our investigation, such as the lack of cell culture experimental confirmation, a relatively small sample size and no follow-up information, it may be assumed that BANCR activity should be explained in a context-dependent manner. The relative abundance of distinct miRs in the tissue could explain the tissue-specific effects of BANCR. It might be that BANCR is a lncRNA with bidirectional effects on the pathogenesis of thyroid cancer. The diversity and tissue-specificity of its targets, as well as the presence of specific mutations that alter BANCR actions, and other confounding factors are all potential reasons for such effects that should be explored further.

To conclude, understanding the mechanisms underlying PTC progression can help to develop more effective treatments and enhance the risk stratification of PTC patients. In this study, we assessed the correlations between molecular markers, like BRAFV600E, BANCR, miR-203a-3p and miR-204-3p, and clinicopathological characteristics of the PTC patients. Our findings show that PTC patients with the BRAFV600E mutation and either high BANCR or miR-203a-3p levels are at high risk of extrathyroidal invasion occurrence, whereas PTC patients with the BRAFV600E mutation and mutually high BANCR and miR-203a-3p levels are not. On the other hand, in the BRAFV600E-negative PTC, the presence of both low miR-204-3p and upregulated BANCR increases the occurrence of thyroid gland capsule invasion. BANCR might act as a sponge for miR-203a-3p and miR-204-3p in PTC, but it seems that the interactions depend on the BRAFV600E mutation presence. Bioinformatic analysis predicted complementary binding between miR-203a-3p/204-3p and BANCR. These discoveries enhance our comprehension of the underlying molecular mechanisms that might influence the aggressive behavior of PTC. They offer a partial description of the intricate interplay of interactions that may occur during the progression of PTC, providing some new perceptions into potential approaches for PTC patients’ management.

## 5. Conclusions

The interplay among BRAFV600E, BANCR, miR-203a-3p and miR-204-3p might influence the development and progression of PTC. According to our results, PTC patients with the BRAFV600E mutation and individually high BANCR or miR-203a-3p levels belong to the high-risk group, whereas PTC patients with the BRAFV600E mutation and mutually high BANCR and miR-203a-3p levels do not. On the other hand, it seems that low miR-204-3p levels and upregulated BANCR work together to increase the aggressiveness of the BRAFV600E-negative PTC. The co-occurrence of tested factors in PTC may provide a new approach for selecting and treating patients with PTC.

## Figures and Tables

**Table 1:** Primers’ sequences.

Method	Primer	Sequence
MASA	BRAF-fw_a (for wild type)	5′-GTGATTTTGGTCTAGCTACAGT-3′
	BRAF-fw_b (for BRAFV600E)	5′-GTGATTTTGGTCTAGCTACAGA-3′
	BRAF-rv	5′-GGCCAAAAATTTAATCAGTGGA-3′
RT-PCR	miR-u6-RT	5′-GTCGTATCCAGTGCAGGGTCCGAGGTATTCGCACTGGATACG ACAAAAATATGG-3′
	miR-203a-3p-RT	5′-GTCGTATCCAGTGCAGGGTCCGAGGTATTCGCACTGGATACG ACCTAGTG-3′
	miR-204-3p-RT	5′-GTCGTATCCAGTGCAGGGTCCGAGGTATTCGCACTGGATACG ACACGTCC-3′
qPCR	BANCR-fw	5′-ACAGGACTCCATGGCAAACG-3′
	BANCR-rv	5′-ATGAAGAAAGCCTGGTGCAGT-3′
	GAPDH-fw	5′-GAAGGTGAAGGTCGGAGT-3′
	GAPDH-rv	5′-GAAGATGGTGATGGGATTTC-3′
	universal miR-rv	5′-CCAGTGCAGGGTCCGAGGTAT-3′
	miR-U6-fw	5′-GCGGTCGCAAGGATGACACG-3′
	miR-203a-3p-fw	5′-CGGCGGTGTGAAATGTTTAGGAC-3′
	miR-204-3p-fw	5′-GCGGTGCUGGGAAGGCAAAG-3′

PCR: polymerase chain reaction, MASA: mutant allele-specific PCR amplification, RT-PCR: reverse transcription and PCR, qPCR: quantitative PCR. -RT: miR-specific stem-loop RT primer, -fw: forward primer, -rv: reverse primer.

**Table 2:** Descriptive statistics of the relative expression levels of tested miRs.

Tissue	miR	N	Mean	SD	Min	Max	Percentiles		
							25th	50th (Median)	75th
NMT adjacent to the BRAFV600E-positive PTC	miR-203a-3p	55	0.0062	0.0079	0.0001	0.0499	0.0017	0.0041	0.0068
	miR-204-3p	55	0.0029	0.0071	0.0001	0.0400	0.0006	0.0014	0.0022
BRAFV600E-positive PTC	miR-203a-3p	55	0.0048	0.0070	0.0001	0.0408	0.0007	0.0021	0.0061
	miR-204-3p	55	0.0013	0.0023	0.00003	0.0140	0.0002	0.0005	0.0013
NMT adjacent to the BRAFV600E-negative PTC	miR-203a-3p	21	0.0046	0.0046	0.0002	0.0186	0.0015	0.0036	0.0053
	miR-204-3p	21	0.0013	0.0010	0.0000	0.0031	0.0004	0.0012	0.0024
BRAFV600E-negative PTC	miR-203a-3p	21	0.0044	0.0065	0.00002	0.0238	0.0004	0.0020	0.0048
	miR-204-3p	21	0.0166	0.0717	0.0002	0.3294	0.00027	0.0006	0.0012

PTC: papillary thyroid carcinoma, NMT: adjacent non-malignant thyroid tissue, miR: microRNA, N: number of cases, SD: standard deviation, Min: minimum value, Max: maximum value.

**Table 3:** Spearman’s correlations of miRs’ relative expressions with clinicopathological parameters of PTC in dependence of the BRAFV600E mutation presence.

Sample	Parameter		Age	Size	DTI	Ei	ID	Lnm	pT	pTNM
BRAFV600E-negative PTC	miR-203a-3p expression	r	−0.320	0.278	−0.092	0.030	−0.430	0.023	0.154	−0.108
		p	0.157	0.223	0.692	0.897	0.052	0.921	0.504	0.642
		N	21	21	21	21	21	21	21	21
	miR-204-3p expression	r	0.031	0.119	−0.393	−0.452 *	−0.552 **	−0.266	−0.463 *	−0.306
		p	0.893	0.608	0.078	**0.040**	**0.010**	0.244	**0.034**	0.177
		N	21	21	21	21	21	21	21	21
BRAFV600E-positive PTC	miR-203a-3p expression	r	0.131	−0.217	0.311 *	0.282 *	0.020	−0.090	0.049	0.104
		p	0.342	0.112	**0.021**	**0.037**	0.885	0.515	0.721	0.448
		N	55	55	55	55	55	55	55	55
	miR-204-3p expression	r	−0.140	0.000	−0.078	0.052	−0.090	−0.172	−0.094	−0.057
		p	0.307	1.000	0.570	0.708	0.513	0.210	0.493	0.677
		N	55	55	55	55	55	55	55	55

PTC: papillary thyroid carcinoma, r: correlation coefficient, p: *p*-value, statistical significance, N: number of cases, DTI: degree of tumor infiltration, Ei: extrathyroidal invasion, ID: intraglandular dissemination, Lnm: lymph node metastasis, pT: tumor classification, pTNM: pathological tumor–node–metastasis stage. * *p* < 0.05, ** *p* < 0.01, statistically significant results are bolded.

**Table 4:** The association of combined miR-203a-3p/204-3p level of expression and BANCR deregulation with the occurrence of extrathyroidal invasion in the BRAFV600E-positive and BRAFV600E-negative PTC.

PTC	Parameter	Extrathyroidal Invasion (Number of Cases)		*p* Value
		No	Yes	
BRAFV600E negative	miR-203a-3p low and BANCR downregulated	5	1	0.378
	miR-203a-3p low or BANCR downregulated	7	2	
	miR-203a-3p high and BANCR upregulated	3	3	
	miR-204-3p high and BANCR downregulated	6	0	0.033 *
	miR-204-3p low or BANCR upregulated	7	2	
	miR-204-3p low and BANCR upregulated	2	4	
BRAFV600E positive	miR-203a-3p low and BANCR downregulated	18	1	0.009 **
	miR-203a-3p high or BANCR upregulated	19	13	
	miR-203a-3p high and BANCR upregulated	4	0	
	miR-204-3p high and BANCR downregulated	16	7	0.763
	miR-204-3p low or BANCR upregulated	22	6	
	miR-204-3p low and BANCR upregulated	3	1	

*p*-value: statistical significance, * *p* < 0.05, ** *p* < 0.01; statistically significant results are bolded.

## Data Availability

The data presented in this study are available on request from the corresponding author.
